# Association between Blood Urea Nitrogen Level and In-Hospital Mortality in Patients with Acute Myocardial Infarction and Subsequent Gastrointestinal Bleeding

**DOI:** 10.31083/j.rcm2505189

**Published:** 2024-05-23

**Authors:** Fangyi Luo, Xue Chen, Yamei Sun, Jie Zhang

**Affiliations:** ^1^Department of Gastroenterology, Beijing Anzhen Hospital, Capital Medical University, 100029 Beijing, China

**Keywords:** gastrointestinal bleeding, blood urea nitrogen, risk stratification, acute myocardial infarction

## Abstract

**Background:**

Limited studies have explored the association between blood urea nitrogen (BUN) levels and in-hospital mortality in patients with acute myocardial infarction (AMI) and subsequent gastrointestinal bleeding (GIB). Our objective was to explore this correlation.

**Methods:**

276 individuals with AMI and subsequent GIB were retrospectively included between January 2012 and April 2023. The predictive value of BUN for in-hospital mortality was assessed through receiver operating characteristic (ROC) curve. Logistic regression models were constructed to assess the relationship between BUN and in-hospital mortality. Propensity score weighting (PSW), sensitivity and subgroup analyses were used to further explore the association.

**Results:**

Fifty-three (19.2%) patients died in the hospital. BUN levels were higher in non-survivors compared with the survivors [(11.17 ± 6.17) vs (8.09 ± 4.24), *p* = 0.001]. The ROC curve suggested that the optimal cut-off for BUN levels to predict in-hospital mortality was 8.45 mmol/L (AUC [area under the ROC curve] 0.678, 95% confidence interval [CI] 0.595–0.761, *p* < 0.001). Multivariable logistic regression showed that elevated BUN levels (≥8.45 mmol/L) were positively association with in-hospital mortality (odds ratio [OR] 4.01, 95% CI 1.55–10.42, *p* = 0.004). After PSW, sensitivity and subgroup analyses, the association remained significant.

**Conclusions:**

Elevated BUN levels were associated with in-hospital mortality in patients with AMI and subsequent GIB.

## 1. Introduction

Acute myocardial infarction (AMI) is a critical medical emergency related to substantial morbidity, mortality, and the utilization of healthcare resources [1]. Aspirin and a P2Y12 inhibitor are the default antithrombotic strategy after AMI [2]. Although this approach reduces the occurrence of ischemic events, it concurrently elevates the risk of bleeding [3]. Gastrointestinal bleeding (GIB) frequently contributes to hemorrhage in patients with AMI [4]. Research findings indicate GIB rates in the range of 0.87% to 1.5% among patients with AMI, correlating with elevated risks of both early and late adverse clinical outcomes [5, 6, 7, 8]. Giving the unfavorable prognosis of patients with GIB after AMI, identifying predictors for early risk stratification is critical.

Some studies have discussed potential risk factors in this setting [6, 9, 10]. In a retrospective study conducted at a single center, participants diagnosed with non-ST-segment elevation myocardial infarction (NSTEMI) and upper GIB showed that several factors may independently influence in-hospital mortality, including age, peak white blood cell count, minimum platelet count, and peak brain natriuretic peptide levels [10]. Another retrospective study, which included 51 patients with AMI who developed GIB, suggested that a decreased hemoglobin level and a high Killip classification may be the risk factors for in-hospital mortality [6]. However, these studies either involved small sample sizes or were limited to patients with NSTEMI, indicating the necessity of more studies to comprehensively evaluate the predictive value of their findings. Moreover, we speculated whether there are additional risk factors correlate with the early prognosis in patients experiencing AMI and subsequent GIB that have not been explored.

Mounting evidence has linked increased blood urea nitrogen (BUN) levels with poor outcomes in patients with AMI [11, 12, 13, 14]. In this context, BUN is susceptible to the influence of various factors, including renal function, cardiac output, systemic perfusion, and neurohumoral regulation. During an AMI, these factors typically change [13]. Furthermore, considering the breakdown and digestion of blood proteins through the gastrointestinal tract, elevated BUN levels are common in patients with acute GIB, especially upper GIB [15]. The Glasgow-Blatchford scale (GBS) is a fully validated score utilizing BUN as a predictive marker to assess the necessity of clinical intervention in patients with upper GIB [16]. However, the value of BUN in predicting in-hospital mortality among individuals experiencing GIB after AMI remains unclear, especially when considered alongside other established risk factors for clinical outcomes.

Thus, our objective was to explore the correlation between BUN levels and in-hospital mortality in patients with AMI and subsequent GIB.

## 2. Materials and Methods

### 2.1 Study Design and Populations

The present investigation was a single-center retrospective study. Patients diagnosed with AMI and subsequently GIB during the same hospitalization between January 2012 and April 2023 at the Beijing Anzhen Hospital of Capital Medical University (Beijing, China) were retrospectively enrolled. AMI was diagnosed based on the Fourth Universal Definition [17]. GIB was defined as the presence of symptoms of gastrointestinal tract bleeding, including coffee ground emesis, hematemesis, melena, or evidence of bleeding observed during endoscopic examination in the gastrointestinal tract [4]. The inclusion criteria involved the confirmation of AMI complicated by GIB. Patients diagnosed with GIB who developed AMI, individuals only presented with positive fecal occult blood tests, and individuals lacking baseline data were excluded from the research. The study was carried out in compliance with the Declaration of Helsinki, and received approval from the Ethics Committee of Beijing Anzhen Hospital, Capital Medical University (approval number: 2023132X). As this study was retrospective, the requirement for informed consent was waived.

### 2.2 Data Collection

Data collected included demographic characteristics, medical history, admission features, laboratory data, and medical treatments. We obtained laboratory data including BUN from the first test conducted upon admission before any acute intervention [such as percutaneous coronary intervention (PCI), or coronary artery bypass grafting (CABG)] and assayed at the central core laboratory. The estimated glomerular filtration rate (eGFR) was calculated by the currently recommended Chronic Kidney Disease Epidemiology Collaboration (CKD-EPI) equation [18]. The primary outcome was all-cause in-hospital mortality. All participants were followed up for death or discharge using complete inpatient medical records.

### 2.3 Statistical Analysis

Continuous variables are presented as mean ± standard deviation (SD) and were compared using the *t*-test. Categorical variables are presented as number (percentage) and were compared using the chi-squared test. The predictive value of admission BUN for in-hospital mortality was assessed through receiver operating characteristic (ROC) curve. Participants were stratified into two cohorts based on the cut-off value of BUN determined by the Youden index. Cumulative survival across different BUN levels were examined through Kaplan–Meier curves, with group comparison was conducted using the log–rank test.

Furthermore, we employed multivariate logistic regression models to assess the relationship between BUN and in-hospital mortality. Model 1 adjusted for age and sex. Model 2 adjusted for: age, sex, STEMI, intra-aortic balloon pump (IABP), Killip classification, chronic kidney disease (CKD), extracorporeal membrane oxygenation (ECMO), cardiogenic shock, heart rate, systolic blood pressure, albumin, eGFR, thrombolysis, PCI, CABG, continuous renal replacement therapy (CRRT), transfusion, and diuretics. Considering significant differences in certain population characteristics among subjects with different BUN levels using inverse probability of weight (IPW) to calculate propensity score weighting (PSW), PSW-weighted multivariate logistic regression analyses were subsequently developed to further control for confounding variables. Moreover, sensitivity and subgroup analyses were performed using multivariable logistic regression Model 2. The interaction effect between BUN and eGFR was evaluated using the log–likelihood ratio test. Statistical analyses were conducted utilizing SPSS 26.0 (IBM Corporation, Armonk, NY, USA) and R version 4.1.1 (R Foundation for Statistical Computing, Vienna, Austria); differences with a *p* < 0.05 were deemed significant.

## 3. Results

### 3.1. Patient Population and Baseline Characteristics

**Fig. 1.**
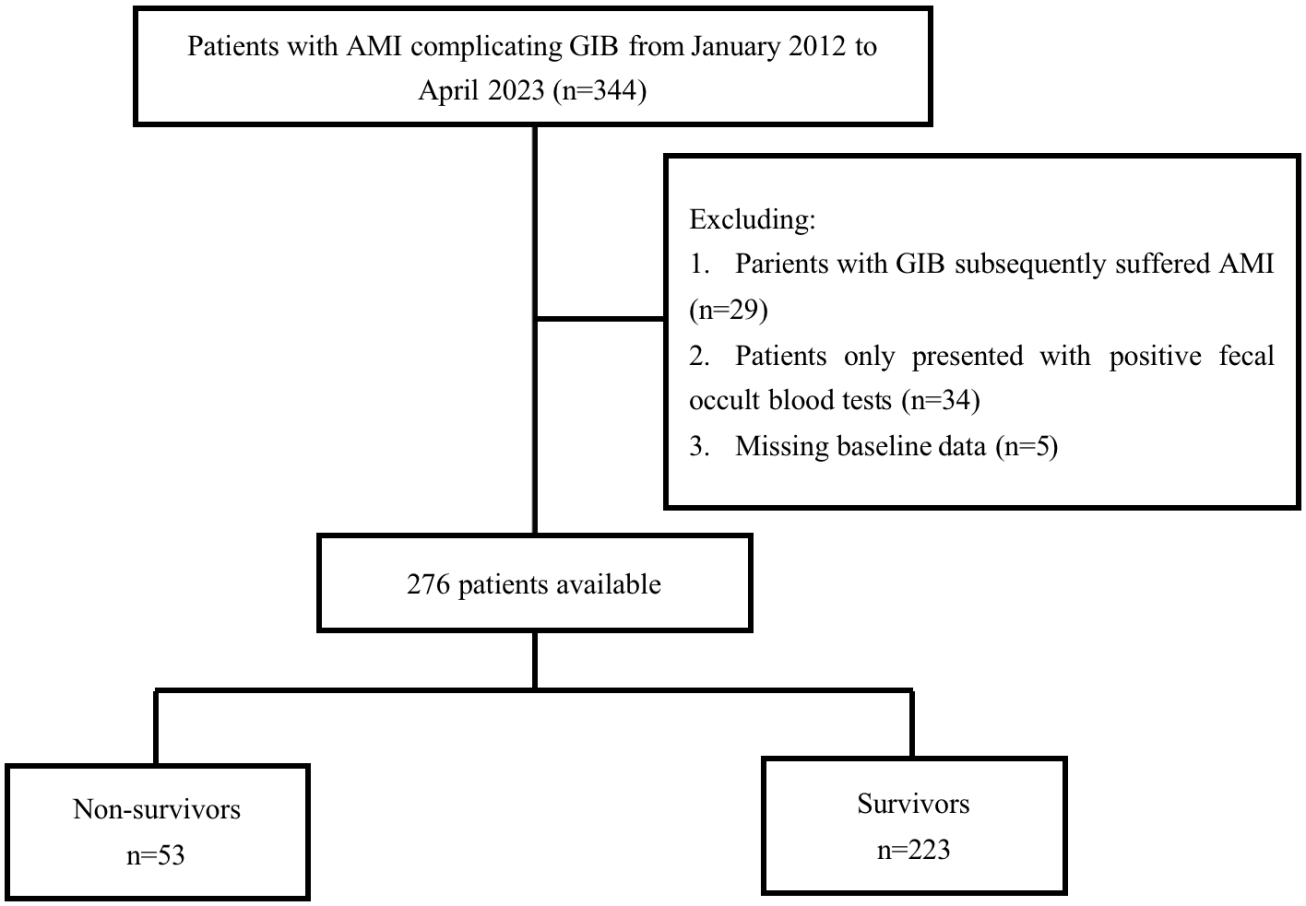
**Flow chart of patient’s selection**. AMI, acute myocardial infarction; GIB, gastrointestinal bleeding.

Between January 2012 and April 2023, 344 patients were diagnosed with AMI complicated by GIB at the authors’ center. After excluding 29 individuals diagnosed with GIB who developed AMI, 34 individuals with only positive fecal occult blood tests, and 5 patients with missing data (Fig. 1), our study included 276 participants. Among the 276 participants enrolled, 184 (66.7%) were male, and the cohort had a mean age of 67.15 ± 11.93 years. Fifty-three (19.2%) patients died in the hospital. Characteristics of the study population stratified by mortality are presented in Table 1. The history of hypertension, diabetes mellitus, CKD, AMI, PCI, CABG, GIB were similar between 2 groups. Compared to the survivors, non-survivors were more likely to have a faster heart rate, clinical evidence of heart failure (Killip classification ≥2) and cardiogenic shock. Moreover, they had lower eGFR and higher BUN levels [(11.17 ± 6.17) vs (8.09 ± 4.24) mmol/L, *p* = 0.001] on admission. In terms of reperfusion therapy, the non-survivor group exhibited a higher preference for CABG over PCI. They were more inclined to receive mechanical circulatory support (such as IABP and ECMO), transfusion and kidney replacement therapy during their hospitalization. In addition, more diuretics and fewer antiplatelet agents were employed in the treatment of non-survivors.

**Table 1.** **Characteristics of study population stratified by mortality**.

Variables		Non-survivors	Survivors	*p*-value
		(n = 53)	(n = 223)	
Age, years		69.49 ± 11.04	66.59 ± 12.09	0.112
Male, n (%)		34 (64.2)	150 (67.3)	0.666
Smoking, n (%)		13 (24.5)	74 (33.2)	0.223
Medical history, n (%)				
	Hypertension	39 (73.6)	148 (66.4)	0.312
	Diabetes mellitus	25 (47.2)	86 (38.6)	0.251
	Chronic kidney disease	11 (20.8)	27 (12.1)	0.101
	History of AMI	10 (18.9)	28 (12.6)	0.231
	History of PCI	12 (22.6)	36 (16.1)	0.262
	History of CAGB	1 (1.9)	10 (4.5)	0.632
	History of GIB	1 (1.9)	14 (6.3)	0.352
Admission features				
	STEMI	37 (69.8)	140 (62.8)	0.337
	Killip classification ≥2	45 (84.9)	112 (50.2)	<0.001
	Cardiogenic shock	19 (35.8)	32 (14.3)	<0.001
	Heart rate >100, beats/min	15 (28.3)	14 (6.3)	<0.001
	Systolic BP <100, mmHg	12 (22.6)	32 (14.3)	0.138
	Hemoglobin <100, g/L	11 (20.8)	40 (17.9)	0.635
	Albumin <30, g/L	9 (17.0)	17 (7.6)	0.067
	eGFR <60, mL/min/1.73 $m^{2}$	32 (60.4)	60 (26.9)	<0.001
	BUN, mmol/L	11.17 ± 6.17	8.09 ± 4.24	0.001
	PT prolongation >3, s	5 (9.4)	15 (6.7)	0.698
Medical treatments, n (%)				
	Thrombolysis	1 (1.9)	13 (5.8)	0.408
	PCI	12 (22.6)	97 (43.5)	0.005
	CAGB	9 (17.0)	8 (3.6)	0.001
	IABP	15 (28.3)	21 (9.4)	<0.001
	ECMO	6 (11.3)	2 (0.9)	<0.001
	CRRT	17 (32.1)	6 (2.7)	<0.001
	Endoscopy	3 (5.7)	16 (7.2)	0.929
	Transfusion	25 (47.2)	51 (22.9)	<0.001
	Aspirin	36 (67.9)	187 (83.9)	0.008
	Clopidogrel or ticagrelor	41 (77.4)	208 (93.3)	<0.001
	Anticoagulants	43 (81.1)	159 (71.3)	0.146
	PPIs	52 (98.1)	223 (100)	0.433
	Diuretics	47 (88.7)	133 (59.6)	<0.001
	ACE inhibitor/ARB	32 (60.4)	141 (63.2)	0.700
	Length of hospital stay, days	12.77 ± 10.29	13.29 ± 9.86	0.736

GIB, gastrointestinal bleeding; CRRT, continuous renal replacement therapy; PCI, percutaneous coronary intervention; BUN, blood urea nitrogen; AMI, acute myocardial infarction; CABG, coronary artery bypass grafting; eGFR, estimated glomerular filtration rate; STEMI, ST-segment elevation myocardial infarction; PT, prothrombin time; IABP, intra-aortic balloon pump; BP, blood pressure; ACE, angiotensin converting enzyme; ECMO, extracorporeal membrane oxygenation; ARB, angiotensin-II receptor blocker; PPIs, proton pump inhibitors.

### 3.2 BUN Level and Mortality

**Fig. 2.**
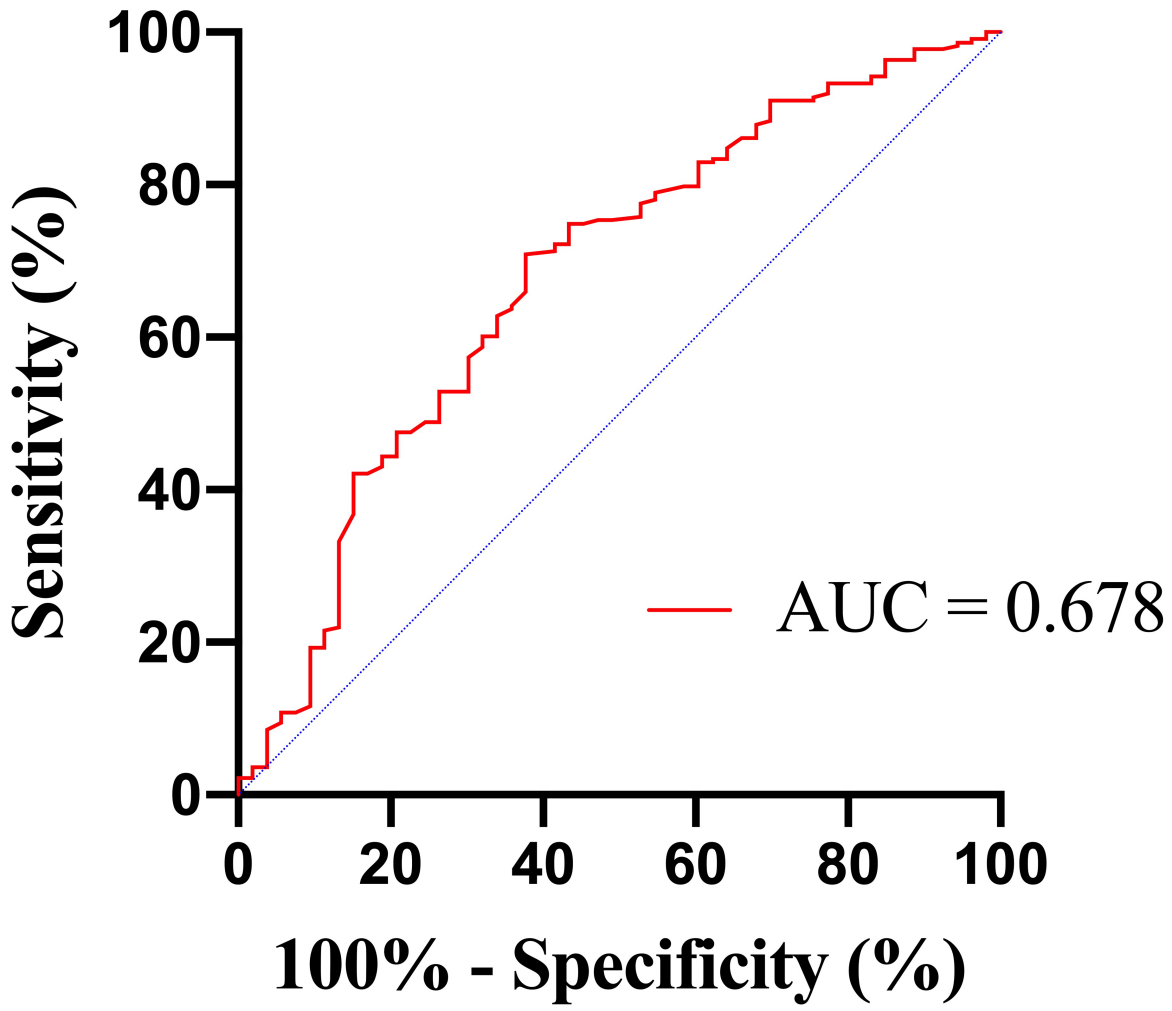
**Receiver operating characteristic (ROC) curves of BUN to predict in-hospital mortality**. BUN, blood urea nitrogen; AUC, area under the ROC curve.

The area under the ROC curve (AUC) for BUN in predicting in-hospital mortality was 0.678 (95% confidence interval [CI] 0.595–0.761; *p* < 0.001) (Fig. 2). The optimal cut–off value of BUN levels for predicting in-hospital mortality in AMI patients with subsequent GIB was determined using Youden index, with a level of 8.45 mmol/L (sensitivity, 62.3%; specificity, 70.9%). **Supplementary Table 1** provides the characteristics and comparisons among patients at different BUN levels. There were 98 patients with BUN ≥8.45 mmol/L and 178 patients with BUN <8.45 mmol/L. Patients exhibiting higher BUN levels had more serious conditions on admission, and had a higher in-hospital mortality (33.7% vs 11.2%, *p* < 0.001).

**Fig. 3.**
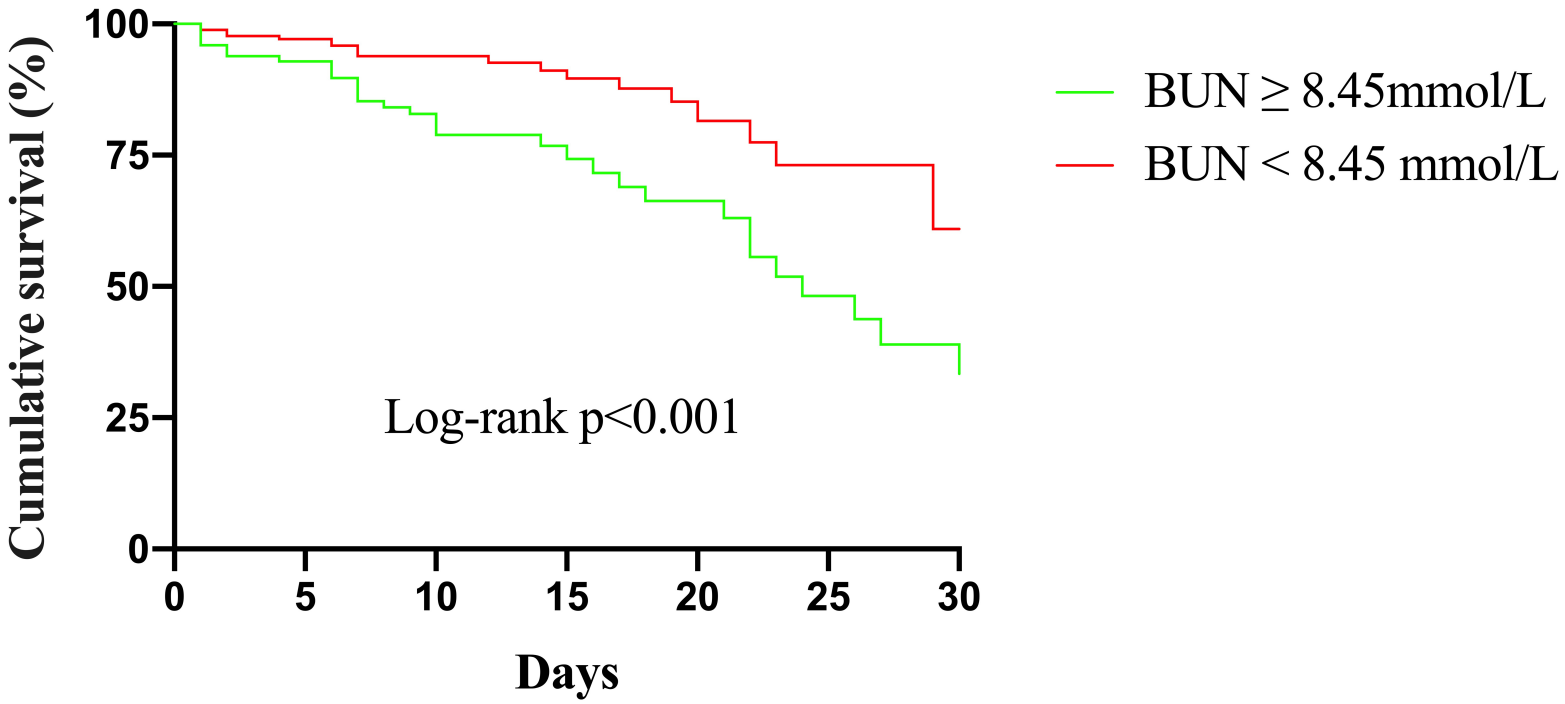
**Kaplan–Meier survival from in-hospital mortality for patients according to different BUN levels**. BUN, blood urea nitrogen.

Kaplan–Meier curves for different BUN levels are presented in Fig. 3. The cumulative in-hospital mortality for patients with BUN ≥8.45 mmol/L was significantly higher than that for patients with BUN <8.45 mmol/L (*p* < 0.001 [log-rank]).

The logistic regression models and the results are shown in Table 2. Elevated BUN levels showed a positive correlation with in-hospital mortality in the unadjusted model, and this association persisted in the adjusted models. Upon incorporating age and sex information in Model 1, increased BUN levels were positively correlated with in-hospital mortality both as a continuous variable (odds ratio [OR] 1.11, 95% CI 1.05–1.18, *p* < 0.001) and as a categorical variable with BUN ≥8.45 mmol/L compared to BUN <8.45 mmol/L (OR 3.84, 95% CI 2.03–7.27, *p* < 0.001). In the fully adjusted Model 2, the higher BUN levels remained associated with in-hospital mortality as a categorical variable (OR 4.01, 95% CI 1.55–10.42, *p* = 0.004).

**Table 2.** **Association of BUN and in-hospital mortality using logistic regression**.

	Unadjusted		Model 1		Model 2	
	Crude OR (95% CI)	*p*-value	Adjusted OR (95% CI)	*p*-value	Adjusted OR (95% CI)	*p*-value
BUN	1.12 (1.06–1.18)	<0.001	1.11 (1.05–1.18)	<0.001	1.06 (0.96–1.17)	0.226
BUN ≥8.45 mmol/L	4.01 (2.15–7.50)	<0.001	3.84 (2.03–7.27)	<0.001	4.01 (1.55–10.42)	0.004

Model 1 was adjusted for age, sex; Model 2 was adjusted for age, sex, CKD, STEMI, Killip classification, cardiogenic shock, heart rate, systolic BP, albumin, eGFR, thrombolysis, PCI, CABG, IABP, ECMO, CRRT, transfusion, and diuretics. OR, odds ratio; CI, confidence interval; BUN, blood urea nitrogen; CKD, chronic kidney disease; STEMI, ST-segment elevation myocardial infarction; BP, blood pressure; eGFR, estimated glomerular filtration rate; PCI, percutaneous coronary intervention; CABG, coronary artery bypass grafting; IABP, intra-aortic balloon pump; ECMO, extracorporeal membrane oxygenation; CRRT, continuous renal replacement therapy.

There were significant differences in population characteristics among patients with different BUN levels in **Supplementary Table 1**. To further control for confounding variables, we also carried out PSW in the study cohort. The comparison of patient characteristics after weighting is available in **Supplementary Table 2**. Baseline characteristics were well balanced. PSW-weighted regression models and the results are presented in Table 3. BUN as a continuous variable showed independently correlation with in-hospital mortality in the unadjusted model (OR 1.12, 95% CI 1.06–1.18, *p* < 0.001), the adjusted Model 1 (OR 1.12, 95% CI 1.06–1.18,* p* < 0.001) and the fully-adjusted Model 2 (OR 1.11, 95% CI 1.02–1.19, *p* = 0.012). Similar findings were obtained when treating BUN as a categorical variable. Compared to BUN <8.45 mmol/L, BUN ≥8.45 mmol/L showed a positive correlation with in-hospital mortality in the non-adjusted model (OR 2.28, 95% CI 1.44–3.61, *p* < 0.001), minimally-adjusted Model 1 (OR 2.25, 95% CI 1.42–3.56, *p* = 0.001), and fully-adjusted Model 2 (OR 4.73, 95% CI 2.41–9.29, *p* < 0.001).

**Table 3.** **Association of BUN and in-hospital mortality using propensity score weighted regression**.

	Unadjusted		Model 1		Model 2	
	Crude OR (95% CI)	*p*-value	Adjusted OR (95% CI)	*p*-value	Adjusted OR (95% CI)	*p*-value
BUN	1.12 (1.06–1.18)	<0.001	1.12 (1.06–1.18)	<0.001	1.11 (1.02–1.19)	0.012
BUN ≥8.5 mmol/L	2.28 (1.44–3.61)	<0.001	2.25 (1.42–3.56)	0.001	4.73 (2.41–9.29)	<0.001

Model 1 was adjusted for age, sex; Model 2 was adjusted for age, sex, CKD, STEMI, Killip classification, cardiogenic shock, heart rate, systolic BP, albumin, eGFR, thrombolysis, PCI, CABG, IABP, ECMO, CRRT, transfusion, and diuretics. OR, odds ratio; CI, confidence interval; BUN, blood urea nitrogen; CKD, chronic kidney disease; STEMI, ST-segment elevation myocardial infarction; BP, blood pressure; eGFR, estimated glomerular filtration rate; PCI, percutaneous coronary intervention; CABG, coronary artery bypass grafting; IABP, intra-aortic balloon pump; ECMO, extracorporeal membrane oxygenation; CRRT, continuous renal replacement therapy.

We performed sensitivity and subgroup analyses to evaluate the persistence of correlation between BUN levels and in-hospital mortality in patients without severe renal insufficiency. After excluding 38 patients with a history of chronic renal insufficiency, elevated BUN levels remain positive association with in-hospital mortality, both as a continuous variable (OR 1.18, 95% CI 1.03–1.36, *p* = 0.018) and as a categorical variable (OR 4.22, 95% CI 1.55–11.49, *p* = 0.005). When patients undergoing regular hemodialysis and those receiving CRRT during hospitalization were excluded (n = 24), BUN ≥8.45 mmol/L had an independent association with in-hospital mortality (OR 4.56, 95% CI 1.64–12.63, *p* = 0.004), whereas this association was not significant when BUN was considered as a continuous variable. Furthermore, subgroup analysis revealed a robust correlation between BUN ≥8.45 mmol/L and in-hospital mortality only in participants with eGFR ≥60 mL/min/1.73 $m^{2}$ (OR 7.27, 95% CI 1.71–30.99, *p* = 0.007). However, it is worth noting that no significant interaction was found between BUN and eGFR (*p* for interaction = 0.990).

## 4. Discussion

Our study revealed an association between BUN levels and the risk for in-hospital mortality in patients with AMI and subsequent GIB, irrespective of established clinical characteristics. Elevated BUN levels may serve as a convenient marker of adverse outcomes in patients experiencing GIB after AMI.

GIB is a serious condition linked to an increased risk for early poor clinical prognosis in patients with AMI [5]. Therefore, one of the most critical challenges for healthcare professionals is to identify patients at high risk of mortality after AMI complicated by GIB. However, there are still many unknown factors that affect in-hospital outcomes in these patients. Some single-center retrospective studies have discussed potential risk factors for this situation; however, BUN has not been found to have a significant impact on in-hospital mortality in individuals with AMI complicated by GIB [6, 9, 10]. To our knowledge, the present study is one of the largest investigations involving AMI patients who developed GIB and reports a higher degree of certainty than previous studies. Moreover, it is the first study to focus on the prognostic effect of BUN in this patient population.

BUN is a metabolic protein product, the concentration of which depends on the balance between renal reabsorption and excretion. For decades, BUN has been considered as an indicator of renal function and prognostic factor in many clinical conditions. The GBS is a commonly used clinical score that incorporates BUN on admission as one of its risk factors. It is designed to predict the necessity for clinical intervention of acute upper GIB [16]. In addition to the GBS, several novel scoring systems using BUN as a predictor have been developed to assist physicians in dealing with upper GIB or lower GIB [19, 20]. In patients with AMI, BUN has demonstrated promising potential as a more important indicator for adverse prognosis rather than creatinine [14]. Furthermore, BUN has been reported to be superior to other renal markers in evaluating the risk for heart failure or even cardiogenic shock [11, 21].

In the present study, after adjusting for other relevant clinical covariates using PSW-weighted multivariate logistic regression models, BUN exhibited a significant association with in-hospital mortality, both as a continuous variable and a categorical variable. We additionally conducted sensitivity and subgroup analyses to evaluate the robustness of this correlation. Sensitivity and subgroup analyses revealed that BUN ≥8.45 mmol/L could still predict in-hospital mortality in patients without severe renal dysfunction. These findings suggest that in patients with AMI who experience subsequent GIB, BUN levels on admission were associated with in-hospital mortality. The current findings can provide valuable insights for initial risk stratification and prognostic evaluation, which seems more useful in patients without severe kidney disease.

The reasons for the correlation between BUN and early prognosis in individuals who develop GIB after AMI can be explained as followed. During the acute stage of myocardial infarction, systemic and renal hypoperfusion are common. These conditions lead to the activation of the sympathetic nervous system and the renin-angiotensin-aldosterone system, which enhance the reabsorption of urea in the proximal tubules [11, 12, 22, 23], resulting in an elevation in BUN levels. This provides additional prognostic information beyond eGFR. Our study observed elevated BUN levels in patients with a higher Killip classification. This may indirectly reflect more severe hemodynamic alterations and neurohormonal activation in conditions of low cardiac output and decompensated heart failure, ultimately leading to an increase in BUN levels.

When patients with AMI experience severe GIB, unstable hemodynamics pose a significant threat. Hypovolemia and dehydration may aggravate renal hypoperfusion [24], leading to neurohormonal activation, which in turn enhances urea reabsorption. High BUN levels have been found to be potentially associated with more severe GIB [25]. Our study also indicated that BUN levels were inversely correlated with hemoglobin levels, suggesting that BUN may indirectly reflect the severity of bleeding. Unfavorable hemodynamic conditions may also result in myocardial ischemia and reinfarction. Even mild GIB can induce systemic inflammation in a prothrombotic state, potentially resulting in recurrent ischemic events [6]. Myocardial reinfarction is usually accompanied by severe hemodynamic alterations and renal hypoperfusion with sequential BUN elevation. Therefore, BUN may serve as a simple marker of renal perfusion and neurohormonal activation in patients with rapid hemodynamic alterations, which is valuable in determining the outcomes of patients with GIB post-AMI. In such patients, alterations in eGFR calculated based on creatinine may not be evident or may lag behind the actual changes. Thus, early identification of patients with elevated BUN levels, proactive treatment of GIB, and enhancement of cardiac function and systemic perfusion are crucial for improving the outcomes of patients experiencing GIB after AMI.

This study has several limitations. Firstly, it was a single-center retrospective design with the inherent shortcomings associated with the analysis of pre-recorded data. Second, the patient cohort was relatively small, primarily due to the low incidence of GIB. As such, further studies with larger cohorts are needed. Despite these limitations, this study identifies the significance of BUN as a simple marker for early prognosis in patients with AMI and subsequent GIB.

## 5. Conclusions

Our study demonstrates that BUN levels were associated with in-hospital mortality in patients with AMI and subsequent GIB. This information will be valuable for early risk stratification of individuals experiencing GIB after AMI.

## Data Availability

The data are obtainable on request from the corresponding author in this study. They are not publicly available due to privacy issues.

## Supplementary Material

Supplementary material associated with this article can be found, in the online version, at https://doi.org/10.31083/j.rcm2505189.
