# Supplementary material for: Association between Blood Urea Nitrogen Level and In-Hospital Mortality in Patients with Acute Myocardial Infarction and Subsequent Gastrointestinal Bleeding
Source: Rev Cardiovasc Med. 2024 May 23;25(5):189. doi: 10.31083/j.rcm2505189 (PMC11267187; doi:10.31083/j.rcm2505189)
Supplement: Supplementary file 1 [file 2153-8174-25-5-189-s1.docx]

Supplemental Table 1. Characteristics of patients with GIB post-AMI stratified by BUN levels before IPW

| Variables | BUN ≥ 8.45mmol/L  (n = 98) | BUN < 8.45mmol/L  (n = 178) | *p*-value |
| --- | --- | --- | --- |
| Age, years | 70.63 ± 11.37 | 65.42 ± 11.90 | 0.001 |
| Male, n (%) | 65 (66.3) | 119 (66.9) | 1.000 |
| Smoking, n (%) | 24 (24.5) | 63 (35.4) | 0.084 |
| Medical history, n (%) |  |  |  |
| Hypertension | 73 (74.5) | 114 (64.0) | 0.101 |
| Diabetes mellitus | 56 (57.1) | 55 (30.9) | <0.001 |
| Chronic kidney disease | 33 (33.7) | 5 (2.8) | <0.001 |
| History of AMI | 23 (23.5) | 15 (8.4) | 0.001 |
| History of PCI | 23 (23.5) | 25 (14.0) | 0.070 |
| History of CAGB | 5 (5.1) | 6 (3.4) | 0.702 |
| History of GIB | 5 (5.1) | 10 (5.6) | 1.000 |
| Admission features |  |  |  |
| STEMI | 53 (54.1) | 124 (69.7) | 0.014 |
| Killip classification ≥ 2 | 65 (66.3) | 92 (51.7) | 0.026 |
| Cardiogenic shock | 21 (21.4) | 30 (16.9) | 0.438 |
| Heart rate > 100, beats/min | 14 (14.3) | 15 (8.4) | 0.189 |
| Systolic BP < 100, mmHg | 11 (11.2) | 33 (18.5) | 0.157 |
| Hemoglobin < 100, g/L | 30 (30.6) | 21 (11.8) | <0.001 |
| Albumin < 30, g/L | 14 (14.3) | 12 (6.7) | 0.066 |
| eGFR < 60, mL/min/1.73 m2 | 68 (69.4) | 24 (13.5) | <0.001 |
| PT prolongation > 3, s | 14 (14.3) | 6 (3.4) | 0.002 |
| Medical therapy |  |  |  |
| Thrombolysis | 2 (2.0) | 12 (6.7) | 0.157 |
| PCI | 25 (25.5) | 84 (47.2) | 0.001 |
| CAGB | 4 (4.1) | 13 (7.3) | 0.422 |
| IABP | 10 (10.2) | 26 (14.6) | 0.394 |
| ECMO | 2 (2.0) | 6 (3.4) | 0.798 |
| CRRT | 15 (15.3) | 8 (4.5) | 0.004 |
| Endoscopy | 5 (5.1) | 14 (7.9) | 0.536 |
| Transfusion | 32 (32.7) | 44 (24.7) | 0.204 |
| Aspirin | 73 (74.5) | 150 (84.3) | 0.070 |
| Clopidogrel or ticagrelor | 84 (85.7) | 165 (92.7) | 0.098 |
| Anticoagulants | 62 (63.3) | 140 (78.7) | 0.009 |
| PPIs | 97 (99.0) | 178 (100.0) | 0.762 |
| Diuretics | 75 (76.5) | 105 (59.0) | 0.005 |
| ACE inhibitor/ARB | 60 (61.2) | 113 (63.5) | 0.809 |
| Clinical outcomes |  |  |  |
| In-hospital mortality, n (%) | 33 (33.7) | 20 (11.2) | <0.001 |
| Length of hospital stay, days | 14.29 ± 11.90 | 12.58 ± 8.62 | 0.173 |

AMI, acute myocardial infarction; GIB, gastrointestinal bleeding; PCI, percutaneous coronary intervention; CABG, coronary artery bypass grafting; STEMI, ST-segment elevation myocardial infarction; BP, blood pressure; BUN, blood urea nitrogen; eGFR, estimated glomerular filtration rate; PT, prothrombin time; IABP, intra-aortic balloon pump; ECMO, extracorporeal membrane oxygenation; CRRT, continuous renal replacement therapy; PPI, proton pump inhibitor; ACE, angiotensin converting enzyme; ARB, angiotensin-II receptor blocker.

Supplemental Table 2. Characteristics of patients with GIB post-AMI stratified by BUN levels after IPW

| Variables | BUN ≥ 8.5mmol/L  (n = 277.74) | BUN < 8.5mmol/L  (n = 247.14) | *p*-value |
| --- | --- | --- | --- |
| Age, years | 67.06 ± 10.76 | 66.17 ± 11.42 | 0.652 |
| Male, n (%) | 212.0 (76.3) | 168.5 (68.2) | 0.295 |
| Smoking, n (%) | 91.5 (32.9) | 91.5 (37.0) | 0.665 |
| Medical history, n (%) |  |  |  |
| Hypertension | 198.0 (71.3) | 156.5 (63.3) | 0.364 |
| Diabetes mellitus | 98.8 (35.6) | 82.8 (33.5) | 0.800 |
| Chronic kidney disease | 36.1 (13.0) | 13.2 (5.4) | 0.110 |
| History of AMI | 49.1 (17.7) | 29.8 (12.1) | 0.383 |
| History of PCI | 59.2 (21.3) | 47.6 (19.2) | 0.777 |
| History of CAGB | 16.1 (5.8) | 10.2 (4.1) | 0.644 |
| History of GIB | 19.7 (7.1) | 13.3 (5.4) | 0.689 |
| Admission features |  |  |  |
| STEMI | 179.7 (64.7) | 164.9 (66.7) | 0.809 |
| Killip classification ≥ 2 | 152.3 (54.8) | 131.8 (53.3) | 0.875 |
| Cardiogenic shock | 72.9 (26.2) | 52.5 (21.3) | 0.575 |
| Heart rate > 100, beats/min | 32.7 (11.8) | 26.7 (10.8) | 0.881 |
| Systolic BP < 100, mmHg | 63.1 (22.7) | 48.4 (19.6) | 0.727 |
| Hemoglobin < 100, g/L | 47.5 (17.1) | 30.5 (12.3) | 0.363 |
| Albumin < 30, g/L | 17.3 (6.2) | 15.0 (6.1) | 0.946 |
| eGFR < 60, mL/min/1.73 m2 | 89.5 (32.2) | 63.4 (25.7) | 0.400 |
| PT prolongation > 3, s | 20.1 (7.2) | 16.6 (6.7) | 0.904 |
| Medical therapy |  |  |  |
| Thrombolysis | 3.2 (1.2) | 12.8 (5.2) | 0.036 |
| PCI | 100.4 (36.1) | 105.7 (42.8) | 0.486 |
| CAGB | 16.3 (5.9) | 17.0 (6.9) | 0.834 |
| IABP | 41.9 (15.1) | 38.8 (15.7) | 0.930 |
| ECMO | 4.4 (1.6) | 13.3 (5.4) | 0.167 |
| CRRT | 31.2 (11.2) | 23.3 (9.4) | 0.789 |
| Endoscopy | 30.8 (11.1) | 17.1 (6.9) | 0.476 |
| Transfusion | 74.1 (26.7) | 66.0 (26.7) | 0.999 |
| Aspirin | 226.7 (81.6) | 202.1 (81.8) | 0.980 |
| Clopidogrel or ticagrelor | 250.5 (90.2) | 220.2 (89.1) | 0.823 |
| Anticoagulants | 198.1 (71.3) | 187.4 (75.8) | 0.572 |
| PPIs | 276.7 (99.6) | 247.1 (100.0) | 0.360 |
| Diuretics | 196.3 (70.7) | 156.9 (63.5) | 0.422 |
| ACE inhibitor/ARB | 162.9 (58.7) | 154.1 (62.4) | 0.694 |

AMI, acute myocardial infarction; GIB, gastrointestinal bleeding; PCI, percutaneous coronary intervention; CABG, coronary artery bypass grafting; STEMI, ST-segment elevation myocardial infarction; BP, blood pressure; BUN, blood urea nitrogen; eGFR, estimated glomerular filtration rate; PT, prothrombin time; IABP, intra-aortic balloon pump; ECMO, extracorporeal membrane oxygenation; CRRT, continuous renal replacement therapy; PPI, proton pump inhibitor; ACE, angiotensin converting enzyme; ARB, angiotensin-II receptor blocker.
